# Recent origin and semi-permeable species boundaries in the scleractinian coral genus *Stylophora* from the Red Sea

**DOI:** 10.1038/srep34612

**Published:** 2016-10-07

**Authors:** Roberto Arrigoni, Francesca Benzoni, Tullia I. Terraneo, Annalisa Caragnano, Michael L. Berumen

**Affiliations:** 1 Red Sea Research Center, Division of Biological and Environmental Science and Engineering, King Abdullah University of Science and Technology, Thuwal 23955-6900, Saudi Arabia; 2 Department of Biotechnology and Biosciences, University of Milano-Bicocca, Piazza della Scienza 2, Milano 20126, Italy; 3 UMR ENTROPIE (IRD, Université de La Réunion, CNRS), Laboratoire d’Excellence-CORAIL, centre IRD de Nouméa, 101 Promenade Roger Laroque, BP A5, 98848 Noumea Cedex, New Caledonia

## Abstract

Reticulate evolution, introgressive hybridisation, and phenotypic plasticity have been documented in scleractinian corals and have challenged our ability to interpret speciation processes. *Stylophora* is a key model system in coral biology and physiology, but genetic analyses have revealed that cryptic lineages concealed by morphological stasis exist in the *Stylophora pistillata* species complex. The Red Sea represents a hotspot for *Stylophora* biodiversity with six morphospecies described, two of which are regionally endemic. We investigated *Stylophora* species boundaries from the Red Sea and the associated *Symbiodinium* by sequencing seven DNA loci. *Stylophora* morphospecies from the Red Sea were not resolved based on mitochondrial phylogenies and showed nuclear allele sharing. Low genetic differentiation, weak isolation, and strong gene flow were found among morphospecies although no signals of genetic recombination were evident among them. *Stylophora mamillata* harboured *Symbiodinium* clade C whereas the other two *Stylophora* morphospecies hosted either *Symbiodinium* clade A or C. These evolutionary patterns suggest that either gene exchange occurs through reticulate evolution or that multiple ecomorphs of a phenotypically plastic species occur in the Red Sea. The recent origin of the lineage leading to the Red Sea *Stylophora* may indicate an ongoing speciation driven by environmental changes and incomplete lineage sorting.

The distributions of several tropical marine species overlap in an area of maximum marine biodiversity located in the Indo-Malay Archipelago known as the Coral Triangle^1^. However, recent works on different reef organisms demonstrated that some peripheral regions, such as the Red Sea^2^,^3^,^4^,^5^ and the Hawaiian Archipelago^4^,^5^,^6^, can act as biodiversity sources, exporting both genetic diversity and morphological novelties^7^. For example, the epicentre of global scleractinian coral diversity is located in the Coral Triangle, but two important and separate centres of biodiversity occur in the Red Sea^3^ and in the subequatorial Western Indian Ocean^8^,^9^. Both these biodiversity centres are characterised by high levels of endemism^3^,^9^,^10^ and, notably, molecular studies have revealed unexpected phylogenetic patterns, unique haplotypes, and endemic taxa that were previously hidden by traditional approaches of species identification based on the study of skeleton morphology^11^,^12^,^13^,^14^,^15^,^16^,^17^,^18^. In particular, the Red Sea harbours a large number of coral species^3^,^8^,^19^,^20^ and the highest level of endemism in the Indian Ocean region^8^. Specifically, 5.5% of the 346 species recorded in the Red Sea are endemic^3^,^21^ whereas levels of endemism are less than 2% for the other areas of the Indian Ocean^8^. The relatively high number of endemic species in the Red Sea may reflect its unusual environmental conditions (e.g., high temperature and salinity^22^) and seems to have multiple origins^21^. Indeed, some taxa diverged from their Indian Ocean counterparts long before the most recent glaciations^21^,^23^ while other taxa may have originated from glacial refugia within the Red Sea^2^,^4^,^5^,^21^.

Accurate evaluations of diversity rely on adequate species identification, a non-trivial issue for corals. Two critical challenges are the presence of cryptic species^14^,^15^,^16^,^24^,^25^,^26^,^27^,^28^, derived from either evolutionary convergence (two non-sister species independently evolve the same phenotype) or morphological stasis (two sister species acquire the same phenotype from a common ancestor), and the occurrence of phenotypic plasticity (the capacity of a single genotype to produce different phenotypes in response to varying environmental conditions)^29^,^30^. Genetic surveys within different coral genera, such as *Stylophora*^14^,^15^,^17^, *Acropora*^16^,^24^,^25^, *Pocillopora*^26^,^27^, and *Seriatopora*^28^, have revealed unexpected cryptic diversity in both sympatric and allopatric populations and over relatively small (e.g., Great Barrier Reef 28, American Samoa^26^, or Western Australia^27^) and relatively large (e.g., Indo-Pacific Ocean^14^,^16^,^17^,^24^) areas. Phenotypic plasticity is a trait often associated with corals and poses many challenges for the reliable identification of these animals^29^. Environmental factors can shape coral colonies and greatly increase intraspecific morphological variation, making the delimitation of species boundaries a difficult task^29^,^30^.

Zooxanthellate scleractinian corals of the genus *Stylophora* are widely distributed and abundant throughout the tropical and sub-tropical coral reef communities of the Indo-Pacific, from the Red Sea to French Polynesia^31^. Their branching coralla and growth forms are highly plastic in relation to environmental gradients dynamically shaping the colonial architecture^32^. *Stylophora* corals are relatively easy to maintain in aquaria and they can produce asexual propagules. The combination of these features has led to the use of *Stylophora* corals, and in particular of *S. pistillata*, as a key model system for scleractinian reproduction^33^, physiology^34^, phenotypic plasticity^32^, coral-dinoflagellate symbiosis^35^, and transcriptome^36^ studies. Nevertheless, recent genetic surveys based on a combination of mitochondrial and nuclear loci have revealed the presence of cryptic divergence and four evolutionary distinct lineages within *S. pistillata* across its entire distribution range^14^,^17^. These data caution that general conclusions arising from comparative investigations of the “lab-rat” *S. pistillata* might be biased by the inclusion of different cryptic entities into experimental designs^14^. Indeed, molecular phylogenies demonstrated the existence of a single homogenous and highly-connected species across the eastern Indian Ocean and the entire Pacific Ocean (clade 1)^14^,^37^, and at least three distinct entities within the western Indian Ocean and the Red Sea (clades 2, 3, and 4)^14^,^15^,^17^. Although the phenotypic plasticity of *S. pistillata* is well documented and greatly contributed to the taxonomic confusion that characterised the genus^31^,^32^, three deeply divergent genetic lineages of *S. pistillata* (clades 1, 2, and 4)^14^ showed similar skeletal morphology and a comparable range of phenotypic variation as result of morphological stasis over a period of 30–50 million years^14^,^17^. On the contrary, clade 3 corresponded unambiguously to *S. madagascarensis* and it is morphologically recognisable from the other three groups at the corallite level^15^. Moreover, differences in associated algal endosymbionts (*Symbiodinium*) were detected among the four lineages^14^, suggesting that different regional environments might influence the ecology of this symbiosis^38^,^39^.

These genetic data corroborated the traditional thought, based on morphological criteria^19^,^20^,^31^, that *Stylophora* displays its peak of diversity in the western and northern Indian Ocean. In particular, *Stylophora* corals from the seas around the Arabian peninsula show remarkable variability in colony morphology and growth form, with a total of six morphospecies assumed to live in this area, namely *S. pistillata, S. subseriata, S. danae, S. kuehlmanni, S. mamillata*, and *S. wellsi*^31^. Interestingly, *S. danae* and *S. kuehlmanni* seem to be endemics of the Gulf of Aden and the Red Sea, whereas *S. mamillata* and *S. wellsi* are restricted to the Red Sea^20^,^31^, suggesting that the ancestral species of the genus *Stylophora* originated in the Red Sea^10^. On the one hand, genetic and morphological data demonstrated that *S. danae, S. kuehlmanni*, and *S. subseriata* from the Gulf of Aden belong to clade 4^14^ and likely represent ecomorphs of *S. pistillata* determined by variation in wave movement and light intensity^15^,^20^. On the other hand, *S. mamillata* and *S. wellsi* display encrusting growth forms with knobby-lobbed verrucae distinguishing them from other *Stylophora* species^19^,^20^ but no genetic data from these two species are available to date. Moreover, *S. mamillata* grows on shaded reef slopes between 20 and 40 m depth, *S. wellsi* occurs in very shallow water of exposed fringing reefs with strong swell and water action^20^, and *S. pistillata* lives in a wide range of habitats and depths^19^. The latter species shows high phenotypic plasticity, exhibiting thick, short branches when growing in shallow, wave-exposed environments and developing slender and anatomising colonies in deeper, protected water^19^,^31^.

In this study, we attempted to assess whether the Red Sea represents a biodiversity hotspot for the coral genus *Stylophora*, integrating new sequence data from this region with previously published phylogenies^14^,^15^,^17^,^37^. A large collection of *Stylophora* samples from different localities along the Saudi Arabian Red Sea (spanning about 2,000 km coastline) was obtained, including colonies from each of the six morphospecies reported to co-occur in the region. Their phylogenetic relationships and the amount of genetic differentiation were investigated, with a particular focus on the Red Sea endemic morphospecies *S. mamillata* and *S. wellsi*^31^. We sequenced three mitochondrial and three nuclear DNA regions previously employed to detect cryptic speciation in *S. pistillata*^14^,^15^,^17^,^37^, as well as one plastid locus from the associated symbiotic dinoflagellates *Symbiodinium*^40^. Our specific aims were to define the genetic boundaries and isolation among *S. mamillata, S. wellsi*, and *S. pistillata* in the Red Sea and to evaluate the timing of the origin of the extant Red Sea *Stylophora* endemics. On the basis of the obtained data, we discuss whether *S. mamillata, S. wellsi*, and *S. pistillata* might represent multiple ecomorphs of a single phenotypically-plastic species or a species complex in the early stages of speciation. The possible roles of phenotypic plasticity, reticulate evolution, introgressive hybridisation, and incomplete lineage sorting are evaluated, and possible causes of the recent diversification of these morphospecies in the Red Sea are proposed.

## Results

### Phylogenetic and haplotype network analyses

**Figure 1:**
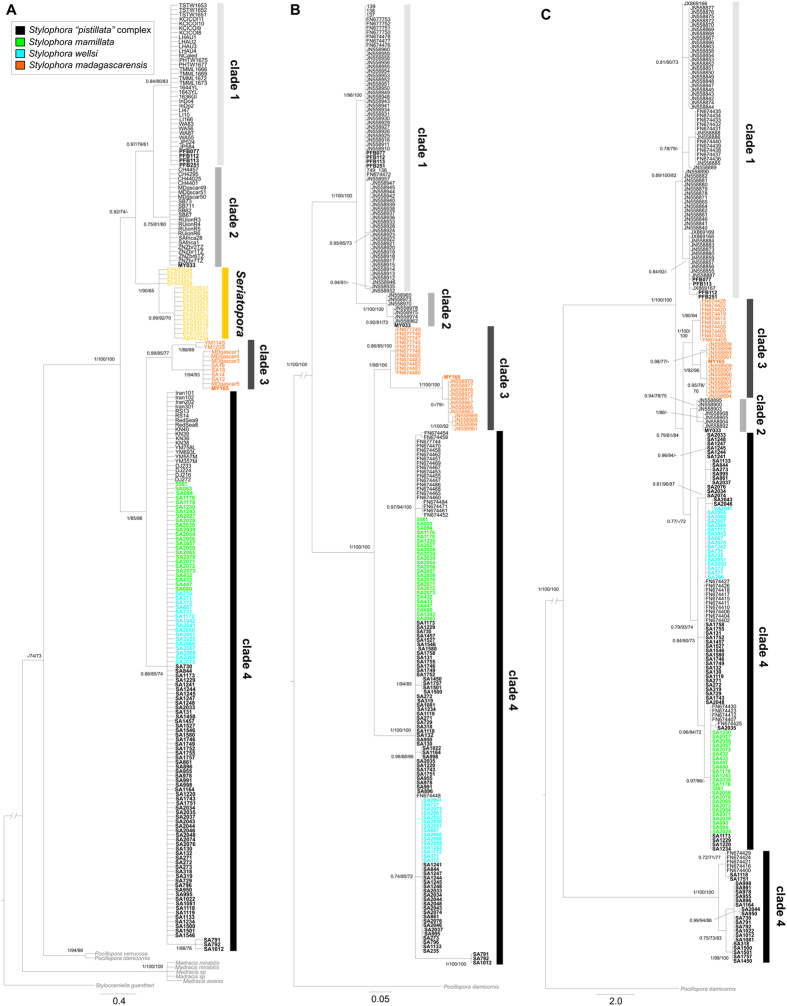
Mitochondrial Bayesian phylogenetic tree reconstructions of the genus *Stylophora*. (**A**) cytochrome oxidase I gene, (**B**) putative control region, (**C**) open reading frame of unknown function. Values at branches represent posterior Bayesian probabilities (>0.7), ML SH-like support (>70%), and MP bootstrap values (>70%), respectively. Dashes (−) indicate nodes that are statistically unsupported. Sequences obtained in this study are indicated in bold. Colours denote *Stylophora* morphospecies as indicated by the embedded key. Clade numbers follow designations of^14^.

We obtained sequence data from three mitochondrial regions, namely the barcoding portion of the cytochrome oxidase I gene (COI), the putative control region (CR), and an open reading frame of unknown function (ORF), along with three nuclear loci, namely the internal transcribed spacer 1 (ITS1) and 2 (ITS2) of ribosomal DNA and the heat shock protein 70 gene (HSP70). Sequences were obtained for each locus from all 103 *Stylophora* colonies sampled across the Red Sea with the exception of three specimens that were not genetically characterised at the ITS1 and ITS2 regions (Tables 1 and 2, Data S1). The single-locus phylogenies based on COI and CR clustered all the analysed *Stylophora* specimens from the Red Sea within clade 4; these further clustered with published sequences of *S. pistillata* from the Gulf of Aden and the Red Sea (Fig. 1). Notably, the COI phylogeny showed that *Stylophora* is not monophyletic due to the inclusion of *Seriatopora* (Fig. 1) as previously reported^14^. The main discrepancy among the three proposed mitochondrial phylogenies was related to the phylogenetic topology inferred from ORF, where clade 4 was split into two main lineages. Of these two lineages, the most divergent one was composed only of colonies from both the Red Sea and the Gulf of Aden identified as belonging to the “*S. pistillata*” complex without any *S. mamillata* and *S. wellsi* samples (Fig. 1C). Mitochondrial haplotype networks suggested that all specimens of *S. mamillata* and *S. wellsi* shared a single ancestral haplotype for the CR locus that was also found in most “*S. pistillata*” complex samples from the Red Sea, while the two former species showed separated haplotypes for the ORF region (Fig. S1). For both mitochondrial loci, sequences of “*S. pistillata*” complex from clade 4 presented several different haplotypes, some of which were highly divergent from the majority of the remaining haplotypes.

**Figure 2:**
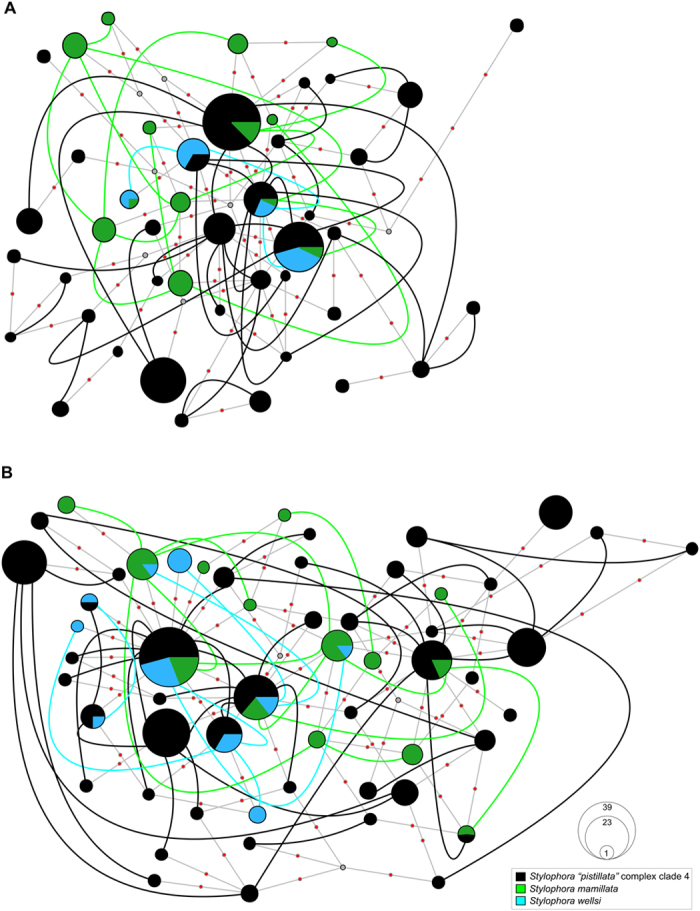
Haplowebs of *Stylophora* morphospecies belonging to clade 4 based on nuclear rDNA. (**A**) ITS1, (**B**) ITS2. Each circle represents a haplotype and its size is proportional to its total frequency. Coloured lines connect haplotypes of heterozygotes individuals and colours denote *Stylophora* morphospecies as indicated by the embedded key. Small grey circles represent missing haplotypes and small orange circles represent a single nucleotide change.

**Figure 3:**
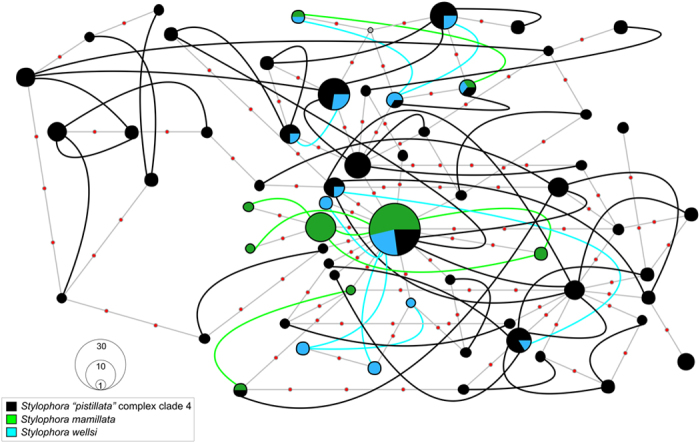
Haplowebs of *Stylophora* morphospecies belonging to clade 4 based on nuclear HSP70 gene. Each circle represents a haplotype and its size is proportional to its total frequency. Coloured lines connect haplotypes of heterozygotes individuals and colours denote *Stylophora* morphospecies as indicated by the embedded key. Small grey circles represent missing haplotypes and small orange circles represent a single nucleotide change.

The phasing of heterozygous nuclear loci was consistent and multisite heterozygotes were resolved with confidence, showing *P* > 0.8 for all the analysed *Stylophora* individuals. We always detected a maximum of two predominant ITS1 and ITS2 sequences in each of the collected specimens, and therefore these two nuclear loci were considered as single-copy nuclear genes (as is HSP70) and each individual was considered either homozygous or heterozygous for these two DNA regions^37^,^41^. Nuclear loci were more diverse than mitochondrial ones in terms of haplotype number, haplotype diversity, and nucleotide diversity (Table 1). Although ITS1^15^,^17^, ITS2^17^, and HSP70 (Fig. S2) resolved *S. pistillata* as a complex of four deeply divergent clades, the nuclear loci grouped the *Stylophora* morphospecies colonies from the Red Sea in a single lineage (clade 4) (Figs 2 and 3). The nuclear haplowebs revealed pervasive allele sharing among *Stylophora* morphospecies from the Red Sea included in clade 4 (Figs 2 and 3). No clear clusters or cohesive groups of individuals sharing a common allele pool were detected across *Stylophora* morphospecies; alleles belonging to each species were highly dispersed across the three nuclear haplowebs. A clear overlap of intra- and interspecific genetic distances was observed in each of the three nuclear loci (Table 3).

### *Symbiodinium* clade association

**Figure 4:**
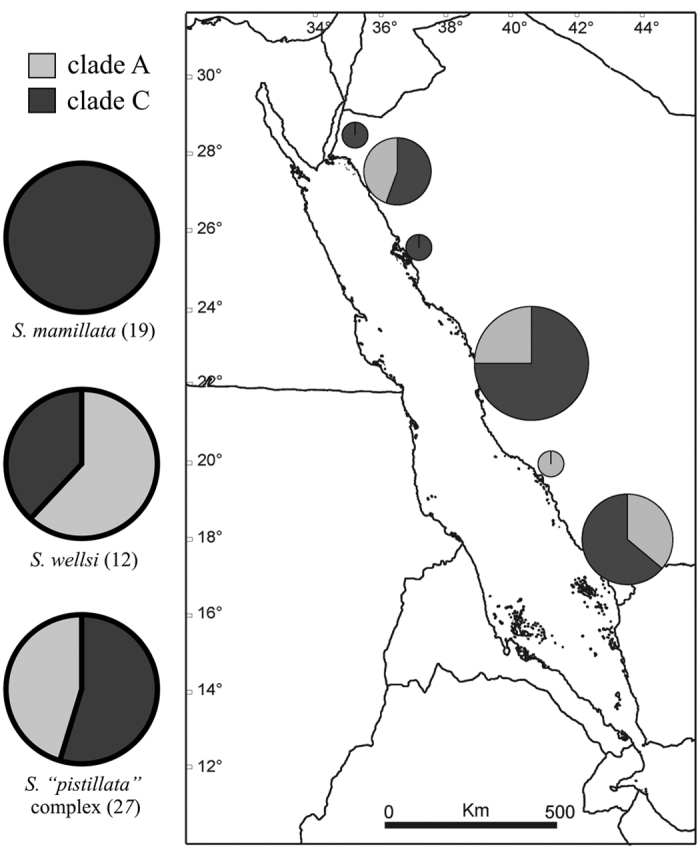
Distribution of *Symbiodinium* clades across the analysed *S. mamillata, S. wellsi*, and “*S. pistillata*” complex (on the left) and across the Red Sea (on the right) based on psbA. Circles in the map refer to the sampling sites of *Stylophora* corals (Data S1) and are proportional to the number of analysed colonies. The circle colours refer to *Symbiodinium* clade A (light grey) and clade C (dark grey). The map was created using Natural Earth (http://www.naturalearthdata.com) and QuantumGIS 2.12 (Quantum GIS Development Team, www.qgis.org).

High-quality *Symbiodinium* sequences of a ~300 bp portion of the plastid psbA gene (psbA) were obtained from a subset of *Stylophora* specimens (n = 64), without showing any signal of polymorphisms^40^. Based on the psbA analysis, all the sampled colonies of *Stylophora* from the Red Sea harboured either *Symbiodinium* clades A or C (Fig. 4). In detail, *Symbiodinium* associated to *S. mamillata* belonged exclusively to clade C. *Stylophora wellsi* and “*S. pistillata*” complex hosted either clades A (n = 22) or C (n = 17), and their *Symbiodinium* composition seemed to be unrelated to the north-south latitudinal gradient of the Red Sea (Fig. 4).

### Population genetic and recombinant analyses

The amount of genetic isolation and differentiation among “*S. pistillata*” complex, *S. mamillata*, and *S. wellsi* was estimated using the hierarchical Analysis of Molecular Variance (AMOVA) analysis and pairwise *F*_ST_ comparisons, assuming each species as a single population and using each of the three individual nuclear loci. The AMOVA results showed no genetic structuring and isolation among species for ITS2 and HSP70 whereas *F*_CT_ was significant for ITS1 (Table 4). Indeed, only a small fraction of the genetic variation was explained by the species grouping (19.34% for ITS1, 6.29% for ITS2, and 9.12% for HSP70); most variance occurred within or among individuals (Table 4). Estimated pairwise *F*_ST_ values were not significantly different from 0 in all comparisons except between the “*S. pistillata*” complex and *S. mamillata* based on HSP70 (Table 5). Collectively, the AMOVA analysis and the pairwise *F*_ST_ values suggested that the three *Stylophora* species were genetically indistinguishable from each other and that limited genetic isolation has occurred.

An analysis of potential recombinant events was conducted to test for hybridization signals among the sequenced nuclear loci, but no evidence of recombination was observed in the three analysed nuclear loci.

### Divergence time estimation

**Figure 5:**
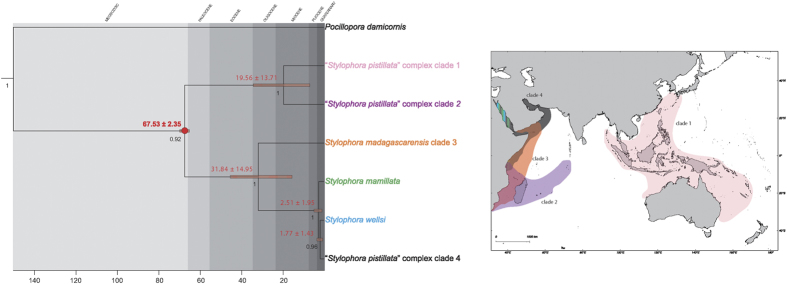
Multi-locus time-calibrated phylogeny reconstruction of the genus *Stylophora* inferred from the concatenated dataset (COI, CR, ORF, ITS1, ITS2, and HSP70) analysed using BEAST. The orange circle marks the node that was time-constrained with fossil (the first appearance of *S. octophyllia* in Santonian and Oman) as described in the text. Values above nodes are mean node ages and orange bars display the 95% highest posterior density (HPD) interval of node ages. Values under nodes are posterior probabilities (>0.9). Clade numbers refer to^14^. Colours in the map are the same as used in the time-calibrated phylogeny on the left. The map was created using Natural Earth (http://www.naturalearthdata.com) and QuantumGIS 2.12 (Quantum GIS Development Team, www.qgis.org).

The multi-locus time-calibrated phylogeny of the genus *Stylophora* was inferred from the concatenated mitochondrial and nuclear datasets for a total of 5,410 bp (Fig. 5). Divergence times between the main *Stylophora* clades and their 95% highest posterior density (HPD) intervals were estimated using the earliest fossil record of the genus from Santonian and Oman, *i*.*e*. the appearance of *Stylophora octophyllia* 65.5–70 Ma^42^,^43^. The group containing the three *Stylophora* species from the Red Sea (“*S. pistillata*” complex, *S. mamillata*, and *S. wellsi*) was estimated to be the most recent lineage within the entire genus, originating 2.51 Ma (95% HPD: 0.56–4.46 Ma). Finally, the estimated divergence times between clades 1 and 2 and between clades 3 and 4 were 19.56 ± 13.71 Ma and 31.84 ± 14.95 Ma, respectively.

## Discussion

**Figure 6:**
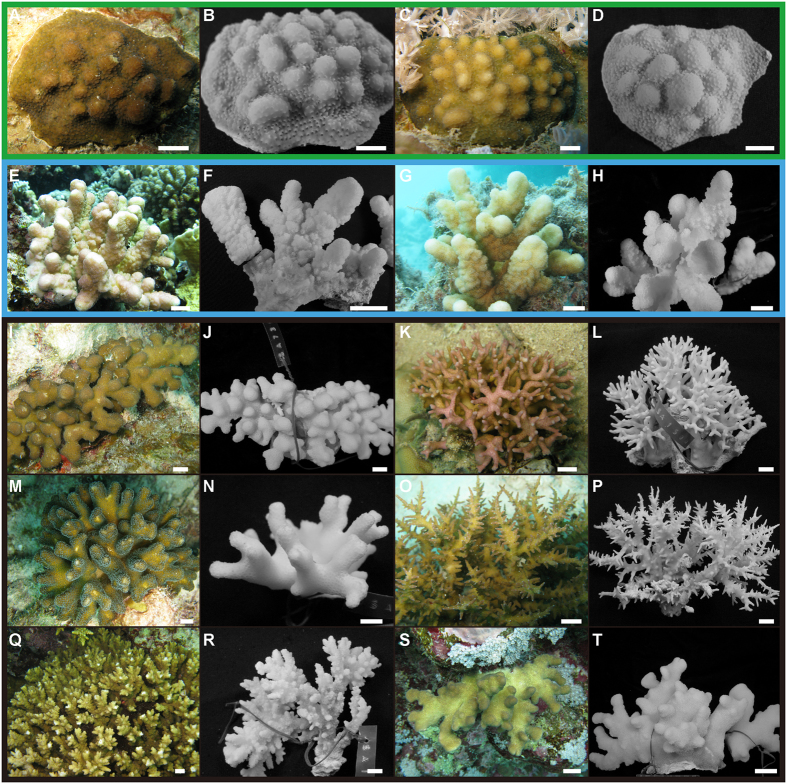
Variability of *in-vivo* colony appearance and skeleton morphology of *Stylophora* morphospecies from the Red Sea. (**A**–**D**) *S. mamillata* SA432 and SA433, (**E**–**H**) *S. wellsi* SA371 and SA731, (**I**–**T**) “*S. pistillata*” complex: (**I**,**J**) *S. danae* SA730, (**K**,**L**) *S. subseriata* SA1500, (**M**,**N**) *S. pistillata* SA1743, (**O**,**P**) *S. kuehlmanni* SA1757, (**Q**,**R**) *S. pistillata* SA1546, showing verrucae typical of *Pocillopora*, (**S**,**T**) *S. pistillata* “*mordax*” form SA1241. Colours refer to *S. mamillata* (green), *S. wellsi* (blue), and “*S. pistillata*” complex (black). Scale bars 1 cm.

In this study we evaluated the importance of the Red Sea as a biodiversity hotspot for the coral genus *Stylophora*, including specimens representing all six morphospecies reported to occur in sympatry in this region (Fig. 6)^19^,^31^. In contrast to expectations based on traditional taxonomy^19^,^31^, the molecular data reported here suggested the presence of a single highly-connected genetic unit of *Stylophora* in this region. These results contradicted the assumption that the Red Sea represents a biodiversity hotspot for *Stylophora* and supported the western Indian Ocean as centre of diversity and origin^9^,^10^, given the co-occurrence of at least three deeply divergent genetic entities in the latter area^14^,^17^. Five DNA markers (COI, CR, ITS1, ITS2, and HSP70) gave congruent results and confirmed the presence of four genetically isolated clades in *Stylophora* across its entire geographic distribution^14^. However, all *Stylophora* corals from the Red Sea belonged to a single molecular clade together with samples from the Gulf of Aden^15^, and the Red Sea morphospecies were indistinguishable on the basis of these five variable and phylogenetically informative loci (COI, CR, ITS1, ITS2, and HSP70). Conversely, the phylogenetic topology inferred from ORF partitioned *S. pistillata* specimens from the Red Sea and the Gulf of Aden in two non-sister clades (Fig. 1C). The discordance among mitochondrial DNA markers has been previously discussed^15^ and is potentially caused by pseudogenes in the mitochondrial genome of some corals, as similarly detected in fish^44^. This scenario may also explain the high values of haplotype and nucleotide diversity in comparison to those found in the other mitochondrial and nuclear loci (Table 1). In fact, genetic diversity among the analysed *Stylophora* morphospecies from the Red Sea (the interspecific genetic distances based on ITS1, ITS2, and HSP70) was among the lowest ever documented in corals, <1% in all pairwise comparisons^45^. Notably, although the Red Sea endemic species *S. mamillata* and *S. wellsi* are easily recognisable based on colony morphology and distinct depth distributions^19^,^20^,^31^, extensive gene flow occurred between these two morphospecies and with *S. pistillata*. Finally, the time-calibrated phylogeny of the genus *Stylophora* demonstrated that the clade leading to the Red Sea morphospecies originated recently, *i*.*e*. 2.51 Ma (95% HPD: 0.56–4.46 Ma).

The incongruence between morphological species delimitations and genetic species boundaries is striking and may be caused by several factors. The collected colonies of *S. pistillata, S. danae, S. subseriata*, and *S. kuehlmanni* exhibit a smooth morphological continuum with regard to the skeletal features traditionally used to identify these taxa^19^,^31^. For example, the branch thickness, the presence of corallite hood, and the coenosteum formation can extensively vary on a single corallum and can be shaped by environmental conditions. Considering the absence of interspecific genetic differentiation based on the six molecular loci employed in this study^14^,^15^,^16^,^17^,^24^,^25^,^26^,^27^,^28^,^37^, the latter three morphospecies should be considered as junior synonyms of *S. pistillata*, as suggested in previous studies^15^,^20^. The Red Sea is characterised by strong latitudinal gradients in environmental variables (*e*.*g*., temperature, salinity, and nutrients)^22^, by a great diversity of habitats and reefs^3^, and by a complex geological history^21^,^46^. These aspects, combined with the extreme phenotypic plasticity^29^,^32^ and habitat generalisation^33^,^34^ documented in *S. pistillata*, can possibly explain the outstanding phenotypic polymorphism of this species in the region.

Despite the lack of genetic differentiation, *S. mamillata* and *S. wellsi* show distinct colony morphologies and depth-partitioning^19^,^20^,^31^. There are at least two possible scenarios that could explain semi-permeable species boundaries among “*S. pistillata*” complex, *S. mamillata*, and *S. wellsi. Stylophora mamillata* and *S. wellsi* may be regional ecomorphs of the highly phenotypic plastic *S. pistillata* or they may actually be valid species. If the latter, they remain connected through genetic exchange or they may be examples of recent speciation events. The first hypothesis (*i*.*e*., *S. mamillata* and *S. wellsi* are ecomorphs of *S. pistillata*) is supported by the extreme phenotypic plasticity of *S. pistillata* in the Red Sea^29^,^32^. A single genotype can produce different phenotypes in response to changing environmental and selective regimes^30^, resulting in distinct morphologies influenced by depth, light, and wave action. For example, a translocated colony of *P. meandrina* began to grow with a morphology more similar to *P. damicornis* after several months^47^; similar translocation experiments could be carried out for *S. mamillata* and *S. wellsi*. Colony morphology is known to be a misleading character in the identification of several corals^11^,^13^,^17^,^24^,^19^,^31^. The encrusting *S. mamillata* is usually characterised by nodes thought to be incipient branches, but these can sometimes grow into small clear branches. Similarly, the formation of verrucae is one of the morphological features prescribed to diagnostically identify *S. wellsi* among *Stylophora* species, but some analysed colonies of *S. pistillata* show this peculiar structure (Fig. 6). Under the second scenario (*i*.*e*., *S. mamillata* and *S. wellsi* are valid species), reticulate evolution through hybridisation and gene exchange or incomplete lineage sorting due to recent speciation may explain the genetic data. Although no recombinant events were detected in the obtained nuclear sequences, introgressive hybridisation and gene exchange have been extensively documented in closely related scleractinian corals. This can be promoted by extensive sympatry and simultaneous multispecies spawning^24^,^48^,^49^,^50^,^51^,^52^. Interestingly, a case of hybridisation between *Pocillopora damicornis* and *S. pistillata* was demonstrated in an isolated island of Australia^48^, as well as among *Pocillopora* species in the Pacific Ocean^50^. These findings suggest that prezygotic isolating mechanisms in the pocilloporids are permeable and may also provide chances for introgression in *Stylophora*. whose morphospecies in the Red Sea may form a *Stylophora* syngameon (a group of species connected through genetic exchange). Moreover, it is widely accepted that hybridisation is enhanced in isolated and peripheral regions^7^,^51^,^52^, such as the Red Sea^3^,^21^, and novel hybrids may have advantageous reproductive abilities^48^. The second process (incomplete lineage sorting) is supported by the time-calibrated phylogeny of *Stylophora*. The phylogenetic reconstruction dated the diversification of the extant Red Sea morphospecies to 2.51 Ma (95% HPD: 0.56–4.46 Ma), matching the period of the establishment of the Red Sea reef fauna^21^. During this time of the Pliocene and Pleistocene (3–4 Ma)^21^, extreme environmental variations occurred, such as fluctuations in temperature and salinity^22^. Moreover, the divergence times between clades 1 and 2 (19.56 ± 13.71 Ma) and between clades 3 and 4 (31.84 ± 14.95 Ma) are in agreement with the evidence of multiple centres of origins for Indian Ocean corals occurring in the Palaeogene and Neogene Tethys, as hypothesised based on the geological events and the distribution of coral endemism in this area^9^,^10^. Therefore, the obtained time-calibrated phylogeny strengthens the hypothesis that suggested the isolation of distinct *Stylophora* populations as a result of the fragmentation of the Tethys Sea, which promoted a great diversification of the genus in the Indian Ocean^3^.

On the basis of these results, *S. mamillata, S. wellsi*, and *S. pistillata* may represent a species complex undergoing early speciation that still shares most ancestral alleles and polymorphisms. The rapid speciation of the three recent morphospecies of *Stylophora* might have been promoted by the strong environmental changes encountered in the Red Sea during Pliocene and Pleistocene^21^,^46^, which may have favoured niche partitioning and ecological differentiation. Furthermore, the extreme phenotypic plasticity of *S. pistillata* might have played a crucial role in promoting speciation, creating intraspecific variations that can form the basis for interspecific diversification^30^. Furthermore, although no genetic isolation was detected among “*S. pistillata*” complex, *S. mamillata*, and *S. wellsi*, they might remain recognisable because disruptive selection may be occurring^53^ or because hybridisation is not pervasive^5^. Indeed, the three morphospecies are ecologically differentiated, showing distinct habitat preference and depth partitioning and, in this condition, disruptive selection can contribute to the maintenance of ecological differences among species and increased phenotypic variation^49^,^53^.

Coevolution of the coral host and symbiont dinoflagellate *Symbiodinium* may play a significant role in niche specialisation, habitat partitioning, and ecological diversification of corals. It may also promote speciation events^54^,^55^. Previous studies on brooding corals of the genera *Madracis* and *Agaricia* across a large depth gradient (2–60 m depth) demonstrated that their associated *Symbiodinium* consistently revealed patterns of host specificity and depth-based zonation^49^,^56^, shaping host bathymetric distribution and ecology. *Stylophora* corals in the Red Sea hosted either *Symbiodinium* clades A or C and, interestingly, all the “deep-water specialist” *S. mamillata* colonies harboured exclusively *Symbiodinium* clade C, whereas the “shallow-water specialist” *S. wellsi* and the generalist “*S. pistillata*” complex were associated with *Symbiodinium* clades A and C along the entire Saudi Arabian Red Sea (spanning 13° of latitude from the Gulf Aqaba to the Farasan Islands). Previous investigations of *S. pistillata* from the Red Sea demonstrated associations with *Symbiodinium* clades A, C, or A + C combinations^14^ and, in particular, it shifts from hosting mainly *Symbiodinium* clade A in shallow waters (2–6 m depth) to *Symbiodinium* clade C in deeper waters (24–26 m depth) in the Gulf of Aqaba (northern Red Sea)^35^. Nevertheless, *Symbiodinium* data presented in this study are not exhaustive and further detailed analyses based on ITS2 typing via next generation sequencing approaches are needed^57^ to clarify if there is a specific association between *S. mamillata* and *Symbiodinium* clade C, which may suggest adaptation of this symbiosis to deep water (below 20 m depth).

## Conclusions

Despite the presence of distinct colony morphologies, *Stylophora* corals from the Red Sea belong to a single cohesive molecular lineage and host less genetic variability compared to other regions, such as the western Indian Ocean and the Gulf of Aden, where up to three genetic clades occur in sympatry^14^,^15^,^17^. These results raise several questions concerning the evolution of the extant *Styophora* morphospecies in the Red Sea. Further analyses are needed in order to evaluate whether *S. mamillata* and *S. wellsi* represent either valid endemic species arising from recent speciation or whether they are simply local ecomorphs of the common “*S. pistillata*” complex adapted to distinct depth and light conditions. Indeed, single and multi-gene approaches may be affected by the slow evolution rate of coral mitochondrial DNA^58^ and by the incomplete concerted evolution of rDNA^59^, resulting in low genetic variation levels of the analysed loci. In these cases, the application of reduced genome approaches, such as RNA-seq or RAD-tag seq, will provide a genome-wide perspective and may improve the phylogenetic resolution and species boundaries definitions, as already demonstrated in the scleractinian coral *Pocillopora*^44^ and the octocoral *Chrysogorgia*^60^. A closer investigation of the reproductive modes in *S. mamillata* and *S. wellsi* may provide insights into the possible occurrence of reproductive barriers and the role of hybridisation events among these morphospecies. Finally, translocation experiments of *S. mamillata* and *S. wellsi* in different depths and environmental regimes will enhance the understanding of phenotypic plasticity and polymorphism whereas associated transcriptomic analyses might indicate which genes are involved in these mechanisms.

## Methods

### Coral collection and identification

A total of 106 colonies of *Stylophora* corals were collected along the coast of the Saudi Arabian Red Sea, between 1–40 m depth. Furthermore, four specimens of *S. pistillata* from Papua New Guinea (clade 1), one colony of *S. pistillata* from Madagascar (clade 2), and one sample of *S. madagascarensis* from Madagascar (clade 3) were included in the analyses. Each colony was photographed underwater and tagged (Data S1). A small portion of tissue (~2 cm^3^) was preserved in 95% ethanol for molecular analyses while the remaining portion (~10 cm^3^ of the colony) was bleached in sodium hypochlorite, rinsed with fresh water, and air-dried. Morphospecies identification was achieved by examining type material and reference monographs^19^,^20^,^31^. For analyses, we considered *S. pistillata, S. subseriata, S. danae*, and *S. kuelhmanni* as part of a single lineage (indicated in the text as “*S. pistillata*” complex), corresponding to *Stylophora* morphs M and L^15^, and clade 4^14^. Indeed, these four species in the Red Sea “*form a smooth continuum with regard to those skeletal structures which have been used previously to help establish them*”^20^. On the contrary, *S. mamillata* and *S. wellsi* are easily morphologically distinguishable from the above four morphospecies, and were therefore treated as separate entities^20^ (Fig. 6).

### DNA extraction and PCR amplifications

Genomic DNA was extracted using the DNeasy Blood & Tissue kit (Qiagen, Hilden, Germany) and DNA concentration of extracts was quantified using a Nanodrop 1000 spectrophotometer (Thermo Scientific, Wilmington, DE, USA). A total of six loci were amplified and sequenced for the analysed *Stylophora* morphospecies^14^,^15^,^17^,^37^: COI, CR, and ORF from the mitochondrial genome, and ITS1, ITS2, and HSP70 gene from nuclear DNA. *Symbiodinium* clades of *Stylophora* hosts were identified using the plastid psbA minicircle (psbA)^40^. The list of primers and PCR annealing temperature is indicated in Table S1. Amplifications were performed in a 12.5 μl PCR reaction mix containing 0.2 μM of each primer, 1X Multiplex PCR Master Mix (Qiagen, Hilden, Germany), and <0.1 ng DNA. PCR consisted of an initial denaturation at 95°C for 15 min, followed by 30 cycles of denaturation at 94 °C for 30 sec, annealing for 1 min, extension at 72 °C for 1 min, and a final extension at 72 °C for 10 min. All PCR products were purified with Illustra ExoStar (GE Healthcare, Buckinghamshire, UK) and directly sequenced in both directions using an ABI 3130xl Genetic Analyzer (Applied Biosystems, Carlsbad, CA, USA). All sequences generated as part of this study were deposited in the EMBL database (Data S1).

### Phase determination, sequence alignment, and recombination assessment

Forward and reverse sequences were assembled and edited using Sequencher 5.3 (Gene Codes Corp., Ann Arbor, MI, USA). Most *Stylophora* colonies showed double peaks and intra-individual polymorphisms from nuclear loci and were thus considered to be heterozygotes^41^. Nuclear sequences were phased using SeqPHASE^61^ and Phase^62^ when alleles showed the same length (n = 21 for ITS1, n = 12 for ITS2, and n = 62 for HSP70), and using Champuru^63^ if the two predominant alleles were of different length (n = 40 for ITS1 and n = 68 for ITS2). In the former case, the two alleles with the highest probability (an order of magnitude greater than the other sequence pairs) were chosen whenever there were multiple possible phases. No obvious or significant differences of genetic diversity and haploweb inference were obtained using alternative phases (results not shown). Alleles of different length were detected only in the ITS1 and ITS2 regions. Phased heterozygotes were represented by both alleles in the further alignments and population genetic analyses. Alignments for each individual locus were performed using MAFFT 7.130b^64^ and the iterative refinement method E-INS-i. Determination of potential recombinant events that can be interpreted as significant signals of hybridisation was carried out using RPD4^65^ for each of the three nuclear loci. In particular, the algorithms RDP, GENECONV, BootScan, MaxChi, Chimaera, SiScan, LARD, and 3SEQ were investigated using the default settings in all cases.

### Phylogenetic and haplotype network analyses

General statistics concerning the obtained sequences and the variability of the seven employed markers were calculated with DnaSP 5.10.1^66^, as reported in Tables 1 and 2. The best evolutionary model for each individual molecular locus was selected using jModeltTest 2.1.1^67^, as indicated in Table S1. Phylogenetic analyses were conducted under three criteria (Bayesian inference (BI), maximum likelihood (ML), and maximum parsimony (MP)) for each of the three individual mitochondrial regions (COI, CR, and ORF) and the nuclear HSP70 gene. For BI analyses, four Markov Chain Monte Carlo (MCMC) chains were run for 8 million generations in MrBayes 3.2.1^68^, saving a tree every 1,000 generations. The analyses were stopped when the deviation of split frequencies was less than 0.01 and all parameters were checked in Tracer 1.6^69^ for effective sampling size and unimodal posterior distribution. The first 25% trees sampled were discarded as burn-in following indications by Tracer 1.6. ML topology was reconstructed using PhyML 3.0^70^ using the Shimodaira and Hasegawa test (SH-like) to check the support of each internal branch. MP analysis was performed using PAUP 4.0b10^71^ and a heuristic search strategy with tree-bisection-reconnection (TBR) branch swapping for 100 replicates and random stepwise addition. Node support was assessed throughout 1,000 bootstrap replicates. The median-joining network analysis implemented in Network 4.613^72^ was applied to each of the three nuclear datasets and to the highly variable mitochondrial CR and ORF regions in order to evaluate relationships among haplotypes. In order to find groups of individuals sharing a common allele pool, nuclear haplonets were converted into haplowebs^73^ by drawing additional connections between the two haplotypes co-occurring in heterozygous individuals using Network Publisher 2.0.0.1 (Fluxus Technology, Suffolk, UK).

### Population genetic analyses

A hierarchical Analysis of Molecular Variance (AMOVA) was performed using Arlequin 3.5.1.2^74^ to determine the percentage of genetic variance explained by morphospecies clustering and the significance of population structure among and within the analysed *Stylophora* morphospecies (subdivided as “*S. pistillata*” complex, *S. mamillata*, and *S. wellsi*), assuming each of the three morphospecies as a population. The genetic differentiation among taxa was estimated by means of pairwise *F*_ST_ with Arlequin 3.5.1.2, calculated with Slatkin’s distance and using genetic distances corrected by a Kimura two-parameter evolutionary model. Significance was tested using 1,000 permutations and allowing a minimum *P*-value of 0.05. Intra- and interspecific genetic distances were calculated using DnaSP 5.10.1 under a Kimura two-parameter evolutionary model and variance was estimated with 1,000 bootstrap replicates.

### Divergence time estimation

In order to provide a provisional estimate of the divergence time of each of the *Stylophora* clades and of the Red Sea *Stylophora* morphospecies, a time-calibrated phylogenetic hypothesis was inferred under a Bayesian framework using BEAST 1.8.2^75^, based on the complete concatenated mitochondrial and nuclear dataset (5,410 bp). We specified the same six partitions as above with unlinked evolutionary models, an uncorrelated (lognormal) clock model, and a Yule tree prior. The analysis was run for 50 million generations, with a sampling frequency of 1,000. After checking adequate mixing and convergence of all runs with Tracer 1.6^69^, the first 20% trees were discarded as burn-in and a maximum clade credibility chronogram with mean node heights was computed using TreeAnnotator 1.8.2^75^. It is known that *Stylophora* occurred both in the Caribbean and the Indo-Pacific during the late Cretaceous but then it disappeared from the former basin during the early Miocene^20^. Because the genus occurs today only in the Indo-Pacific, we time-constrained the node leading to *Stylophora* spp in the tree based on the fossil record of *S. octophyllia*, which first appear in Santonian and Oman during the Maastrichtian around 65.5–70 Mya^43^,^44^.

## Additional Information

**How to cite this article**: Arrigoni, R. *et al*. Recent origin and semi-permeable species boundaries in the scleractinian coral genus *Stylophora* from the Red Sea. *Sci. Rep.* **6**, 34612; doi: 10.1038/srep34612 (2016).

## Supplementary Material

Supplementary Information

## Figures and Tables

**Table 1:** Summary of information for the sequenced markers of *Stylophora* morphospecies belonging to clade 4 and the relative substitution models used in the phylogenetic analyses.

	N_C_	N_S_	H	S	S_V_	S_PI_	H_D_	N_D_	Substitution model
COI	103	103	2	546	3	3	0.048 ± 0.026	0.001 ± 0.001	HKY + G
CR	103	103	9	1147	66	56	0.213 ± 0.049	0.004 ± 0.001	GTR + I
ORF	103	103	10	1107	291	290	0.821 ± 0.016	0.092 ± 0.006	GTR + G
ITS1	101	162	45	517	34	34	0.935 ± 0.008	0.008 ± 0.003	SYM + G
ITS2	102	182	56	630	30	28	0.93 ± 0.011	0.005 ± 0.002	GTR + I + G
HSP70	103	165	61	1463	47	43	0.952 ± 0.01	0.003 ± 0.001	HKY + I + G

No. of sequenced colonies (N_C_), No. of obtained sequences (N_S_), No. of unique haplotypes or alleles (H), No. of sites (S), No. of variable sites (%) (S_V_), No. of parsimony-informative sites (%) (S_PI_), Haplotype diversity (H_D_), Nucleotide diversity (N_D_).

**Table 2:** Sample size and molecular diversity indices for *S. mamillata, S. wellsi*, and “*S. pistillata*” complex based on ITS1, ITS2, and HSP70 sequence data.

	ITS1					ITS2					HSP70				
	n	n_s_	n_h_	H_D_	N_D_	n	n_s_	n_h_	H_D_	N_D_	n	n_s_	n_h_	H_D_	N_D_
*S. mamillata*	24	44	13	0.959 ± 0.014	0.008 ± 0.005	24	43	14	0.916 ± 0.029	0.005 ± 0.003	24	35	9	0.694 ± 0.063	0.001 ± 0.001
*S. wellsi*	15	25	4	0.801 ± 0.056	0.005 ± 0.003	15	29	10	0.818 ± 0.066	0.003 ± 0.002	16	26	13	0.911 ± 0.039	0.002 ± 0.002
“*S. pistillata*” complex	101	164	35	0.955 ± 0.008	0.011 ± 0.007	90	147	43	0.955 ± 0.006	0.007 ± 0.005	63	112	50	0.976 ± 0.005	0.004 ± 0.003

No. of sequenced colonies (n), No. of obtained sequences (n_s_), No. of unique haplotypes or alleles (n_h_), Haplotype diversity (H_D_), Nucleotide diversity (N_D_)

**Table 3:** Interspecific and intraspecific average genetic distances expressed as percent (standard deviation in brackets) for *S. mamillata, S. wellsi*, and “*S. pistillata*” complex based on ITS1, ITS2, and HSP70 sequence data.

	ITS1			ITS2			HSP70		
	*S. mamillata*	*S. wellsi*	“*S. pistillata*” complex	*S. mamillata*	*S. wellsi*	“*S. pistillata*” complex	*S. mamillata*	*S. wellsi*	“*S. pistillata*” complex
*S. mamillata*	0.812% (±0.242)			0.407% (±0.158)			0.112% (±0.038)		
*S. wellsi*	0.882% (±0.281)	0.509% (±0.228)		0.387% (±0.124)	0.222% (±0.073)		0.189% (±0.052)	0.232% (±0.063)	
“*S. pistillata*” complex	0.941% (±0.242)	0.737% (±0.219)	0.832% (±0.234)	0.492% (±0.142)	0.417% (±0.119)	0.493% (±0.141)	0.294% (±0.063)	0.343% (±0.067)	0.396% (±0.087)

**Table 4:** Analysis of molecular variance (AMOVA) of the partitioning of genetic variation among *S. mamillata, S. wellsi*, and “*S. pistillata*” complex based on ITS1, ITS2, and HSP70 sequence data.

Locus	Source of variation	d. f.	Sum of squares	Variance components	Percentage of variation	Fixation indices
ITS1	Among species	2	161.938	1.416	19.34	*F*_CT_ = **0**.**193**
	Among individuals within species	117	843.173	2.106	28.77	*F*_SC_ = **0**.**357**
	Within individuals	92	349.503	3.799	51.89	*F*_ST_ = **0**.**481**
ITS2	Among species	2	90.886	0.636	6.29	*F*_CT_ = 0.063
	Among individuals within species	113	1243.552	2.753	27.20	*F*_SC_ = **0**.**291**
	Within individuals	93	625.917	6.731	66.51	*F*_ST_ = 0.**335**
HSP70	Among species	2	26.876	0.236	9.12	*F*_CT_ = 0.091
	Among individuals within species	100	294.836	1.002	38.77	*F*_SC_ = 0.427
	Within individuals	62	83.502	1.347	52.11	*F*_ST_ = 0.479

d. f. = degrees of freedom. The values of fixation indices (*F*_CT_, *F*_SC_, *F*_ST_) in bold are significantly high at *P* < 0.05 (1,000 permutations).

**Table 5:** Pairwise *F*_ST_ values (below diagonal) and their significance as *P*-value (above the diagonal) among *S. mamillata, S. wellsi*, and “*S. pistillata*” complex based on ITS1, ITS2, and HSP70 sequence data.

	ITS1			ITS2			HSP70		
	*S. mamillata*	*S. wellsi*	“*S. pistillata*” complex	*S. mamillata*	*S. wellsi*	“*S. pistillata*” complex	*S. mamillata*	*S. wellsi*	“*S. pistillata*” complex
*S. mamillata*		0.510	0.256		0.075	0.232		0.283	0.033
*S. wellsi*	0.032		0.374	0.028		0.329	0.072		0.412
“*S. pistillata*” complex	0.017	0.022		0.047	0.087		**0**.**119**	0.011	

The values of *F*_ST_ in bold are significantly high at *P* < 0.05 (1,000 permutations).
