# Supplementary material for: Recent origin and semi-permeable species boundaries in the scleractinian coral genus Stylophora from the Red Sea
Source: Sci Rep. 2016 Oct 7;6:34612. doi: 10.1038/srep34612 (PMC5054360; doi:10.1038/srep34612)

Recent origin and semi-permeable species boundaries in the scleractinian coral genus *Stylophora* from the Red Sea

Roberto Arrigoni[1], Francesca Benzoni[2,3], Tullia I. Terraneo[1], Annalisa Caragnano[3] & Michael L. Berumen[1]

[1] Red Sea Research Center, Division of Biological and Environmental Science and Engineering, King Abdullah University of Science and Technology, Thuwal 23955-6900, Saudi Arabia. [2] Department of Biotechnology and Biosciences, University of Milano-Bicocca, Piazza della Scienza 2, Milano 20126, Italy. [3] Institut de Recherche pour le Développement, UMR227 Coreus2, 101 Promenade Roger Laroque, BP A5, 98848 Noumea Cedex, New Caledonia. Correspondence and requests for materials should be addressed to R. A. (email: roberto.arrigoni@kaust.edu.sa)

Supplementary information accompanies this paper at http://www.nature.com/srep

Data S1. List of *Stylophora* specimens analysed for this study with voucher number, *Stylophora* molecular clade, sampling locality and geographic coordinate, collector, and EMBL accession number of each of the seven sequenced loci.

Voucher number	Genus	Species	Molecular clade	Sampling locality	Coordinates	Collector	COI	CR	ORF	ITS1	ITS2	HSP70	psbA
SA1172	*Stylophora*	*wellsi*	4	Shaybarah, Red Sea	N 25°21.694', E 36°54.754'	Benzoni F	LT160970	LT161079	LT161188	LT161297-LT161298	LT161458-LT161459	LT161639	LT161813
SA1242	*Stylophora*	*wellsi*	4	Thuwal, Red Sea	N 22° 32.2077', E 39° 04.173'	Caragnano A	LT160971	LT161080	LT161189	LT161299-LT161300	LT161460	LT161640	LT161814
SA2041	*Stylophora*	*wellsi*	4	Thuwal, Red Sea	N 22° 32.2077', E 39° 04.173'	Terraneo TI	LT160972	LT161081	LT161190	LT161301-LT161302	LT161461-LT161462	LT161641	
SA2050	*Stylophora*	*wellsi*	4	Thuwal, Red Sea	N 22° 32.2077', E 39° 04.173'	Terraneo TI	LT160973	LT161082	LT161191	LT161303-LT161304	LT161463-LT161464	LT161642	
SA2051	*Stylophora*	*wellsi*	4	Thuwal, Red Sea	N 22° 32.2077', E 39° 04.173'	Terraneo TI	LT160974	LT161083	LT161192	LT161305	LT161465-LT161466	LT161643-LT161644	LT161815
SA2052	*Stylophora*	*wellsi*	4	Thuwal, Red Sea	N 22° 32.2077', E 39° 04.173'	Terraneo TI	LT160975	LT161084	LT161193	LT161306-LT161307	LT161467-LT161468	LT161645-LT161646	
SA2066	*Stylophora*	*wellsi*	4	Thuwal, Red Sea	N 22° 32.2077', E 39° 04.173'	Terraneo TI	LT160976	LT161085	LT161194	LT161308-LT161309	LT161469-LT161470	LT161647-LT161648	LT161816

SA2067	*Stylop hora*	*wellsi*	4	Thuwal, Red Sea	N 22° 32.2077', E 39° 04.173'	Terrane o TI	LT16 0977	LT16 1086	LT16 1195			LT161649	LT16 1817
SA2068	*Stylop hora*	*wellsi*	4	Thuwal, Red Sea	N 22° 32.2077', E 39° 04.173'	Terrane o TI	LT16 0978	LT16 1087	LT16 1196	LT161310	LT161471-LT161472	LT161650-LT161651	LT16 1818
SA2069	*Stylop hora*	*wellsi*	4	Thuwal, Red Sea	N 22° 32.2077', E 39° 04.173'	Terrane o TI	LT16 0979	LT16 1088	LT16 1197	LT161311-LT161312	LT161473-LT161474	LT161652-LT161653	LT16 1819
SA2075	*Stylop hora*	*wellsi*	4	Thuwal, Red Sea	N 22° 32.2077', E 39° 04.173'	Terrane o TI	LT16 0980	LT16 1089	LT16 1198	LT161313	LT161475-LT161476	LT161654	LT16 1820
SA235	*Stylop hora*	*wellsi*	4	Farasan Banks, Red Sea	N 20°07.690', E 40°12.513'	Benzoni F	LT16 0981	LT16 1090	LT16 1199	LT161314	LT161477-LT161478	LT161655-LT161656	LT16 1821
SA371	*Stylop hora*	*wellsi*	4	Thuwal, Red Sea	N 22° 32.2077', E 39° 04.173'	Benzoni F	LT16 0982	LT16 1091	LT16 1200	LT161315	LT161479-LT161480	LT161657-LT161658	LT16 1822
SA372	*Stylop hora*	*wellsi*	4	Thuwal, Red Sea	N 22° 32.2077', E 39° 04.173'	Benzoni F	LT16 0983	LT16 1092	LT16 1201	LT161316-LT161317	LT161481-LT161482	LT161659-LT161660	
SA667	*Stylop hora*	*wellsi*	4	Al Wajh, Red Sea	N 25°23.515', E 36°41.035'	Benzoni F	LT16 0984	LT16 1093	LT16 1202	LT161318-LT161319	LT161483-LT161484	LT161661-LT161662	LT16 1823
SA731	*Stylop hora*	*wellsi*	4	Ras Al-Ubayd, Red Sea	N 26°44.167', E 36°02.659'	Benzoni F	LT16 0985	LT16 1094	LT16 1203	LT161320-LT161321	LT161485-LT161486	LT161663-LT161664	LT16 1824
5581	*Stylop hora*	*mamillata*	4	Thuwal, Red Sea	N 22° 32.2077', E 39° 04.173'	Baird AH	LT16 0986	LT16 1095	LT16 1204	LT161322-LT161323	LT161487-LT161488	LT161665-LT161666	LT16 1825
SA093	*Stylop hora*	*mamillata*	4	Farasan Banks, Red Sea	N 20°07.690', E 40°12.513'	Benzoni F	LT16 0987	LT16 1096	LT16 1205	LT161324-LT161325	LT161489-LT161490	LT161667	
SA094	*Stylop hora*	*mamillata*	4	Farasan Banks, Red Sea	N 20°07.690', E 40°12.513'	Benzoni F	LT16 0988	LT16 1097	LT16 1206	LT161326-LT161327	LT161491	LT161668	LT16 1826
SA1176	*Stylop hora*	*mamillata*	4	Shaybarah, Red Sea	N 25°21.694', E 36°54.754'	Benzoni F	LT16 0989	LT16 1098	LT16 1207	LT161328-LT161329	LT161492-LT161493	LT161669	LT16 1827
SA1178	*Stylop hora*	*mamillata*	4	Shaybarah, Red Sea	N 25°21.694', E 36°54.754'	Benzoni F	LT16 0990	LT16 1099	LT16 1208	LT161330	LT161494-LT161495	LT161670	LT16 1828
SA1230	*Stylop hora*	*mamillata*	4	Thuwal, Red Sea	N 22° 32.2077', E 39° 04.173'	Caragna no A	LT16 0991	LT16 1100	LT16 1209	LT161331-LT161332	LT161496-LT161497	LT161671	LT16 1829
SA1243	*Stylop hora*	*mamillata*	4	Thuwal, Red Sea	N 22° 32.2077', E 39° 04.173'	Caragna no A	LT16 0992	LT16 1101	LT16 1210	LT161333-LT161334	LT161498	LT161672-LT161673	
SA2027	*Stylop hora*	*mamillata*	4	Thuwal, Red Sea	N 22° 32.2077', E 39° 04.173'	Terrane o TI	LT16 0993	LT16 1102	LT16 1211	LT161335-LT161336	LT161499-LT161500	LT161674	LT16 1830
SA2028	*Stylop hora*	*mamillata*	4	Thuwal, Red Sea	N 22° 32.2077', E 39° 04.173'	Terrane o TI	LT16 0994	LT16 1103	LT16 1212	LT161337-LT161338	LT161501-LT161502	LT161675-LT161676	LT16 1831
SA2030	*Stylop hora*	*mamillata*	4	Thuwal, Red Sea	N 22° 32.2077', E 39° 04.173'	Terrane o TI	LT16 0995	LT16 1104	LT16 1213	LT161339-LT161340	LT161503	LT161677-LT161678	
SA2039	*Stylop hora*	*mamillata*	4	Thuwal, Red Sea	N 22° 32.2077', E 39° 04.173'	Terrane o TI	LT16 0996	LT16 1105	LT16 1214	LT161341-LT161342	LT161504-LT161505	LT161679	LT16 1832
SA2054	*Stylop hora*	*mamillata*	4	Thuwal, Red Sea	N 22° 32.2077', E 39° 04.173'	Terrane o TI	LT16 0997	LT16 1106	LT16 1215	LT161343-LT161344	LT161506-LT161507	LT161680-LT161681	LT16 1833
SA2056	*Stylop hora*	*mamillata*	4	Thuwal, Red Sea	N 22° 32.2077', E 39° 04.173'	Terrane o TI	LT16 0998	LT16 1107	LT16 1216	LT161345-LT161346	LT161508-LT161509	LT161682-LT161683	LT16 1834
SA2057	*Stylop hora*	*mamillata*	4	Thuwal, Red Sea	N 22° 32.2077', E 39° 04.173'	Terrane o TI	LT16 0999	LT16 1108	LT16 1217	LT161347	LT161510-LT161511	LT161684	LT16 1835

SA2058	*Stylop hora*	*mamillata*	4	Thuwal, Red Sea	N 22° 32.2077', E 39° 04.173'	Terrane o TI	LT16 1000	LT16 1109	LT16 1218	LT161348-LT161349	LT161512-LT161513	LT161685-LT161686	LT16 1836
SA2065	*Stylop hora*	*mamillata*	4	Thuwal, Red Sea	N 22° 32.2077', E 39° 04.173'	Terrane o TI	LT16 1001	LT16 1110	LT16 1219	LT161350	LT161514	LT161687	LT16 1837
SA2070	*Stylop hora*	*mamillata*	4	Thuwal, Red Sea	N 22° 32.2077', E 39° 04.173'	Terrane o TI	LT16 1002	LT16 1111	LT16 1220	LT161351-LT161352	LT161515-LT161516	LT161688-LT161689	LT16 1838
SA2071	*Stylop hora*	*mamillata*	4	Thuwal, Red Sea	N 22° 32.2077', E 39° 04.173'	Terrane o TI	LT16 1003	LT16 1112	LT16 1221	LT161353-LT161354	LT161517-LT161518	LT161690	
SA2072	*Stylop hora*	*mamillata*	4	Thuwal, Red Sea	N 22° 32.2077', E 39° 04.173'	Terrane o TI	LT16 1004	LT16 1113	LT16 1222	LT161355-LT161356	LT161519-LT161520	LT161691	
SA2073	*Stylop hora*	*mamillata*	4	Thuwal, Red Sea	N 22° 32.2077', E 39° 04.173'	Terrane o TI	LT16 1005	LT16 1114	LT16 1223	LT161357-LT161358	LT161521	LT161692	LT16 1839
SA432	*Stylop hora*	*mamillata*	4	Thuwal, Red Sea	N 22° 32.2077', E 39° 04.173'	Benzoni F	LT16 1006	LT16 1115	LT16 1224	LT161359-LT161360	LT161522-LT161523	LT161693-LT161694	LT16 1840
SA433	*Stylop hora*	*mamillata*	4	Thuwal, Red Sea	N 22° 32.2077', E 39° 04.173'	Benzoni F	LT16 1007	LT16 1116	LT16 1225	LT161361-LT161362	LT161524-LT161525	LT161695-LT161696	LT16 1841
SA447	*Stylop hora*	*mamillata*	4	Thuwal, Red Sea	N 22° 32.2077', E 39° 04.173'	Benzoni F	LT16 1008	LT16 1117	LT16 1226	LT161363	LT161526-LT161527	LT161697-LT161698	LT16 1842
SA680	*Stylop hora*	*mamillata*	4	Al Wajh, Red Sea	N 25°23.515', E 36°41.035'	Benzoni F	LT16 1009	LT16 1118	LT16 1227	LT161364-LT161365	LT161528-LT161529	LT161699	LT16 1843
MY033	*Stylop hora*	*"pistillata" complex*	2	Mayotte		Benzoni F	LT16 1010	LT16 1119	LT16 1228			LT161700	LT16 1844
MY165	*Stylop hora*	*"pistillata" complex*	3	Mayotte		Benzoni F	LT16 1011	LT16 1120	LT16 1229			LT161701-LT161702	LT16 1845
PFB077	*Stylop hora*	*"pistillata" complex*	1	Papua New Guinea		Benzoni F	LT16 1012	LT16 1121	LT16 1230			LT161703	LT16 1846
PFB112	*Stylop hora*	*"pistillata" complex*	1	Papua New Guinea		Benzoni F	LT16 1013	LT16 1122	LT16 1231			LT161704	LT16 1847
PFB113	*Stylop hora*	*"pistillata" complex*	1	Papua New Guinea		Benzoni F	LT16 1014	LT16 1123	LT16 1232			LT161705-LT161706	LT16 1848
PFB251	*Stylop hora*	*"pistillata" complex*	1	Papua New Guinea		Benzoni F	LT16 1015	LT16 1124	LT16 1233			LT161707-LT161708	LT16 1849
SA1012	*Stylop hora*	*"pistillata" complex*	4	Gulf of Aqaba, Red Sea	N 28°11.077', E 34°38.287'	Caragna no A	LT16 1016	LT16 1125	LT16 1234	LT161366-LT161367	LT161530-LT161531	LT161709	
SA1022	*Stylop hora*	*"pistillata" complex*	4	Gulf of Aqaba, Red Sea	N 28°11.077', E 34°38.287'	Caragna no A	LT16 1017	LT16 1126	LT16 1235		LT161532-LT161533	LT161710	
SA1081	*Stylop hora*	*"pistillata" complex*	4	Gulf of Aqaba, Red Sea	N 28°11.077', E 34°38.287'	Caragna no A	LT16 1018	LT16 1127	LT16 1236	LT161368-LT161369	LT161534	LT161711-LT161712	
SA1118	*Stylop hora*	*"pistillata" complex*	4	Shaybarah, Red Sea	N 25°21.694', E 36°54.754'	Caragna no A	LT16 1019	LT16 1128	LT16 1237	LT161370-LT161371	LT161535-LT161536	LT161713	LT16 1850
SA1119	*Stylop hora*	*"pistillata" complex*	4	Shaybarah, Red Sea	N 25°21.694', E 36°54.754'	Caragna no A	LT16 1020	LT16 1129	LT16 1238	LT161372-LT161373	LT161537-LT161538	LT161714-LT161715	
SA1133	*Stylop hora*	*"pistillata" complex*	4	Shaybarah, Red Sea	N 25°21.694', E 36°54.754'	Caragna no A	LT16 1021	LT16 1130	LT16 1239	LT161374	LT161539-LT161540	LT161716-LT161717	
SA1164	*Stylop hora*	*"pistillata" complex*	4	Shaybarah, Red Sea	N 25°21.694', E 36°54.754'	Caragna no A	LT16 1022	LT16 1131	LT16 1240	LT161375-LT161376	LT161541	LT161718	LT16 1851
SA1173	*Stylop hora*	*"pistillata" complex*	4	Shaybarah, Red Sea	N 25°21.694', E 36°54.754'	Benzoni F	LT16 1023	LT16 1132	LT16 1241	LT161377-LT161378	LT161542	LT161719-LT161720	

SA1220	*Stylop hora*	*"pistillata" complex*	4	Thuwal, Red Sea	N 22° 32.2077', E 39° 04.173'	Benzoni F	LT16 1024	LT16 1133	LT16 1242	LT161379-LT161380	LT161543-LT161544	LT161721-LT161722	
SA1229	*Stylop hora*	*"pistillata" complex*	4	Thuwal, Red Sea	N 22° 32.2077', E 39° 04.173'	Caragna no A	LT16 1025	LT16 1134	LT16 1243	LT161381	LT161545-LT161546	LT161723-LT161724	
SA1234	*Stylop hora*	*"pistillata" complex*	4	Thuwal, Red Sea	N 22° 32.2077', E 39° 04.173'	Caragna no A	LT16 1026	LT16 1135	LT16 1244	LT161382-LT161383	LT161547-LT161548	LT161725-LT161726	
SA1241	*Stylop hora*	*"pistillata" complex*	4	Thuwal, Red Sea	N 22° 32.2077', E 39° 04.173'	Caragna no A	LT16 1027	LT16 1136	LT16 1245	LT161384-LT161385	LT161549-LT161550	LT161727	LT16 1852
SA1244	*Stylop hora*	*"pistillata" complex*	4	Thuwal, Red Sea	N 22° 32.2077', E 39° 04.173'	Caragna no A	LT16 1028	LT16 1137	LT16 1246	LT161386-LT161387	LT161551-LT161552	LT161728	
SA1245	*Stylop hora*	*"pistillata" complex*	4	Thuwal, Red Sea	N 22° 32.2077', E 39° 04.173'	Caragna no A	LT16 1029	LT16 1138	LT16 1247	LT161388	LT161553	LT161729-LT161730	
SA1247	*Stylop hora*	*"pistillata" complex*	4	Thuwal, Red Sea	N 22° 32.2077', E 39° 04.173'	Caragna no A	LT16 1030	LT16 1139	LT16 1248	LT161389	LT161554-LT161555	LT161731-LT161732	
SA1248	*Stylop hora*	*"pistillata" complex*	4	Thuwal, Red Sea	N 22° 32.2077', E 39° 04.173'	Caragna no A	LT16 1031	LT16 1140	LT16 1249	LT161390-LT161391	LT161556-LT161557	LT161733-LT161734	LT16 1853
SA130	*Stylop hora*	*"pistillata" complex*	4	Farasan Banks, Red Sea	N 20°07.690', E 40°12.513'	Benzoni F	LT16 1032	LT16 1141	LT16 1250	LT161392	LT161558-LT161559	LT161735-LT161736	
SA131	*Stylop hora*	*"pistillata" complex*	4	Farasan Banks, Red Sea	N 20°07.690', E 40°12.513'	Benzoni F	LT16 1033	LT16 1142	LT16 1251	LT161393-LT161394	LT161560-LT161561	LT161737	
SA132	*Stylop hora*	*"pistillata" complex*	4	Farasan Banks, Red Sea	N 20°07.690', E 40°12.513'	Benzoni F	LT16 1034	LT16 1143	LT16 1252	LT161395	LT161562-LT161563	LT161738-LT161739	
SA1450	*Stylop hora*	*"pistillata" complex*	4	Farasan Banks, Red Sea	N 20°07.690', E 40°12.513'	Benzoni F	LT16 1035	LT16 1144	LT16 1253	LT161396-LT161397	LT161564-LT161565	LT161740-LT161741	
SA1457	*Stylop hora*	*"pistillata" complex*	4	Farasan Banks, Red Sea	N 20°07.690', E 40°12.513'	Benzoni F	LT16 1036	LT16 1145	LT16 1254	LT161398	LT161566-LT161567	LT161742-LT161743	
SA1500	*Stylop hora*	*"pistillata" complex*	4	Farasan Banks, Red Sea	N 20°07.690', E 40°12.513'	Benzoni F	LT16 1037	LT16 1146	LT16 1255	LT161399	LT161568	LT161744	
SA1501	*Stylop hora*	*"pistillata" complex*	4	Farasan Banks, Red Sea	N 20°07.690', E 40°12.513'	Benzoni F	LT16 1038	LT16 1147	LT16 1256	LT161400	LT161569-LT161570	LT161745	
SA1527	*Stylop hora*	*"pistillata" complex*	4	Farasan Island, Red Sea	N 16°45.286' E 41°29.552'	Benzoni F	LT16 1039	LT16 1148	LT16 1257	LT161401	LT161571-LT161572	LT161746-LT161747	LT16 1854
SA1546	*Stylop hora*	*"pistillata" complex*	4	Farasan Island, Red Sea	N 16°45.286' E 41°29.552'	Benzoni F	LT16 1040	LT16 1149	LT16 1258	LT161402-LT161403	LT161573-LT161574	LT161748-LT161749	LT16 1855
SA1580	*Stylop hora*	*"pistillata" complex*	4	Farasan Island, Red Sea	N 16°45.286' E 41°29.552'	Benzoni F	LT16 1041	LT16 1150	LT16 1259	LT161404	LT161575-LT161576	LT161750	LT16 1856
SA1743	*Stylop hora*	*"pistillata" complex*	4	Farasan Island, Red Sea	N 16°45.286' E 41°29.552'	Benzoni F	LT16 1042	LT16 1151	LT16 1260	LT161405	LT161577-LT161578	LT161751-LT161752	LT16 1857
SA1746	*Stylop hora*	*"pistillata" complex*	4	Farasan Island, Red Sea	N 16°45.286' E 41°29.552'	Benzoni F	LT16 1043	LT16 1152	LT16 1261	LT161406	LT161579-LT161580	LT161753-LT161754	
SA1749	*Stylop hora*	*"pistillata" complex*	4	Farasan Island, Red Sea	N 16°45.286' E 41°29.552'	Benzoni F	LT16 1044	LT16 1153	LT16 1262	LT161407	LT161581-LT161582	LT161755-LT161756	LT16 1858

SA1751	*Stylophora*	*"pistillata" complex*	4	Farasan Island, Red Sea	N 16°45.286' E 41°29.552'	Benzoni F	LT161045	LT161154	LT161263	LT161408-LT161409	LT161583-LT161584	LT161757-LT161758	LT161859
SA1752	*Stylophora*	*"pistillata" complex*	4	Farasan Island, Red Sea	N 16°45.286' E 41°29.552'	Benzoni F	LT161046	LT161155	LT161264	LT161410	LT161585-LT161586	LT161759-LT161760	
SA1755	*Stylophora*	*"pistillata" complex*	4	Farasan Island, Red Sea	N 16°45.286' E 41°29.552'	Benzoni F	LT161047	LT161156	LT161265	LT161411-LT161412	LT161587-LT161588	LT161761-LT161762	LT161860
SA1757	*Stylophora*	*"pistillata" complex*	4	Farasan Island, Red Sea	N 16°45.286' E 41°29.552'	Benzoni F	LT161048	LT161157	LT161266	LT161413	LT161589-LT161590	LT161763-LT161764	LT161861
SA1758	*Stylophora*	*"pistillata" complex*	4	Farasan Island, Red Sea	N 16°45.286' E 41°29.552'	Benzoni F	LT161049	LT161158	LT161267	LT161414	LT161591-LT161592	LT161765-LT161766	
SA2033	*Stylophora*	*"pistillata" complex*	4	Thuwal, Red Sea	N 22° 32.2077', E 39° 04.173'	Terraneo TI	LT161050	LT161159	LT161268	LT161415	LT161593	LT161767-LT161768	LT161862
SA2034	*Stylophora*	*"pistillata" complex*	4	Thuwal, Red Sea	N 22° 32.2077', E 39° 04.173'	Terraneo TI	LT161051	LT161160	LT161269	LT161416-LT161417	LT161594-LT161595	LT161769-LT161770	LT161863
SA2035	*Stylophora*	*"pistillata" complex*	4	Thuwal, Red Sea	N 22° 32.2077', E 39° 04.173'	Terraneo TI	LT161052	LT161161	LT161270	LT161418-LT161419	LT161596-LT161597	LT161771-LT161772	
SA2037	*Stylophora*	*"pistillata" complex*	4	Thuwal, Red Sea	N 22° 32.2077', E 39° 04.173'	Terraneo TI	LT161053	LT161162	LT161271	LT161420-LT161421	LT161598-LT161599	LT161773-LT161774	
SA2043	*Stylophora*	*"pistillata" complex*	4	Thuwal, Red Sea	N 22° 32.2077', E 39° 04.173'	Terraneo TI	LT161054	LT161163	LT161272	LT161422-LT161423	LT161600-LT161601	LT161775-LT161776	
SA2044	*Stylophora*	*"pistillata" complex*	4	Thuwal, Red Sea	N 22° 32.2077', E 39° 04.173'	Terraneo TI	LT161055	LT161164	LT161273	LT161424-LT161425	LT161602	LT161777-LT161778	
SA2046	*Stylophora*	*"pistillata" complex*	4	Thuwal, Red Sea	N 22° 32.2077', E 39° 04.173'	Terraneo TI	LT161056	LT161165	LT161274	LT161426	LT161603	LT161779	LT161864
SA2048	*Stylophora*	*"pistillata" complex*	4	Thuwal, Red Sea	N 22° 32.2077', E 39° 04.173'	Terraneo TI	LT161057	LT161166	LT161275	LT161427-LT161428	LT161604-LT161605	LT161780	LT161865
SA2074	*Stylophora*	*"pistillata" complex*	4	Thuwal, Red Sea	N 22° 32.2077', E 39° 04.173'	Terraneo TI	LT161058	LT161167	LT161276	LT161429	LT161606-LT161607	LT161781-LT161782	LT161866
SA2076	*Stylophora*	*"pistillata" complex*	4	Thuwal, Red Sea	N 22° 32.2077', E 39° 04.173'	Terraneo TI	LT161059	LT161168	LT161277	LT161430-LT161431	LT161608-LT161609	LT161783	LT161867
SA271	*Stylophora*	*"pistillata" complex*	4	Farasan Island, Red Sea	N 16°45.286' E 41°29.552'	Benzoni F	LT161060	LT161169	LT161278	LT161432	LT161610-LT161611	LT161784-LT161785	
SA272	*Stylophora*	*"pistillata" complex*	4	Farasan Island, Red Sea	N 16°45.286' E 41°29.552'	Benzoni F	LT161061	LT161170	LT161279	LT161433	LT161612	LT161786	
SA273	*Stylophora*	*"pistillata" complex*	4	Farasan Island, Red Sea	N 16°45.286' E 41°29.552'	Benzoni F	LT161062	LT161171	LT161280	LT161434	LT161613-LT161614	LT161787-LT161788	LT161868
SA318	*Stylophora*	*"pistillata" complex*	4	Farasan Island, Red Sea	N 16°45.286' E 41°29.552'	Benzoni F	LT161063	LT161172	LT161281	LT161435	LT161615	LT161789	LT161869
SA319	*Stylophora*	*"pistillata" complex*	4	Farasan Island, Red Sea	N 16°45.286' E 41°29.552'	Benzoni F	LT161064	LT161173	LT161282	LT161436	LT161616-LT161617	LT161790	LT161870
SA729	*Stylophora*	*"pistillata" complex*	4	Ras Al-Ubayd, Red Sea	N 26°44.167', E 36°02.659'	Benzoni F	LT161065	LT161174	LT161283	LT161437	LT161618	LT161791-LT161792	LT161871
SA730	*Stylophora*	*"pistillata" complex*	4	Ras Al-Ubayd, Red Sea	N 26°44.167', E 36°02.659'	Benzoni F	LT161066	LT161175	LT161284	LT161438-LT161439	LT161619-LT161620	LT161793-LT161794	LT161872
SA791	*Stylophora*	*"pistillata" complex*	4	Jaz'air Silah, Red Sea	N 27°38.302', E 35°18.369'	Benzoni F	LT161067	LT161176	LT161285	LT161440-LT161441	LT161622	LT161795-LT161796	
SA792	*Stylophora*	*"pistillata" complex*	4	Jaz'air Silah, Red Sea	N 27°38.302', E 35°18.369'	Benzoni F	LT161068	LT161177	LT161286	LT161442-LT161443	LT161623-LT161624	LT161797-LT161798	

SA796	*Stylop hora*	*"pistillata" complex*	4	Jaz'air Silah, Red Sea	N 27°38.302', E 35°18.369'	Caragna no A	LT16 1069	LT16 1178	LT16 1287		LT161625	LT161799	
SA844	*Stylop hora*	*"pistillata" complex*	4	Jazirat Burcan, Red Sea	N 27°54'35.6", E 35°03'55.5"	Benzoni F	LT16 1070	LT16 1179	LT16 1288	LT161444-LT161445	LT161626-LT161627	LT161800-LT161801	LT16 1873
SA861	*Stylop hora*	*"pistillata" complex*	4	Jazirat Burcan, Red Sea	N 27°54'35.6", E 35°03'55.5"	Caragna no A	LT16 1071	LT16 1180	LT16 1289	LT161446-LT161447	LT161628	LT161802-LT161803	LT16 1874
SA896	*Stylop hora*	*"pistillata" complex*	4	Jazirat Burcan, Red Sea	N 27°54'35.6", E 35°03'55.5"	Caragna no A	LT16 1072	LT16 1181	LT16 1290	LT161448-LT161449	LT161629-LT161630	LT161804	LT16 1875
SA950	*Stylop hora*	*"pistillata" complex*	4	Gulf of Aqaba, Red Sea	N 28°11.077', E 34°38.287'	Caragna no A	LT16 1073	LT16 1182	LT16 1291	LT161450-LT161451	LT161631-LT161632	LT161805	
SA955	*Stylop hora*	*"pistillata" complex*	4	Gulf of Aqaba, Red Sea	N 28°11.077', E 34°38.287'	Caragna no A	LT16 1074	LT16 1183	LT16 1292	LT161452	LT161633-LT161634	LT161806	LT16 1876
SA978	*Stylop hora*	*"pistillata" complex*	4	Gulf of Aqaba, Red Sea	N 28°11.077', E 34°38.287'	Caragna no A	LT16 1075	LT16 1184	LT16 1293	LT161453	LT161621	LT161807-LT161808	
SA991	*Stylop hora*	*"pistillata" complex*	4	Gulf of Aqaba, Red Sea	N 28°11.077', E 34°38.287'	Caragna no A	LT16 1076	LT16 1185	LT16 1294	LT161454	LT161635	LT161809-LT161810	
SA995	*Stylop hora*	*"pistillata" complex*	4	Gulf of Aqaba, Red Sea	N 28°11.077', E 34°38.287'	Caragna no A	LT16 1077	LT16 1186	LT16 1295	LT161455-LT161456	LT161636	LT161811	
SA998	*Stylop hora*	*"pistillata" complex*	4	Gulf of Aqaba, Red Sea	N 28°11.077', E 34°38.287'	Caragna no A	LT16 1078	LT16 1187	LT16 1296	LT161457	LT161637-LT161638	LT161812	

Table S1. List of molecular loci sequenced in this study with their targeted taxon, PCR primer name and sequence, PCR annealing temperature, and the sources used. Refer to Table 1 for more details on individual loci.

Locus	Targeted taxon	Primer name	Sequence (5' – 3')	Annealing temperature (°C)	Source
COI (mitochondrial)	*Stylophora*	LCO1490 HCO2198	GTCAACAAATCATAAAGATATTGG	51	Folmer et al. (1994)
		HCO2198	TAACTTCAGGGTGACCAAAAAATCA	51	Folmer et al. (1994)
CR (mitochondrial)	*Stylophora*	FNAD5.2deg	GCCYAGRGGTGTTGTTCAAT	51.5	Flot et al. (2008a)
		RCOI3deg	CGCAGAAAGCTCBARTCGTA	51.5	Flot et al. (2008b)
ORF (mitochondrial)	*Stylophora*	FATP6.1	TTTGGGSATTCGTTTAGCAG	53	Flot et al. (2008)
		RORF	SCCAATATGTTAAACASCATGTCA	53	Flot et al. (2008)
ITS1 rDNA (nuclear)	*Stylophora*	F18S1	CGATYGAAYGGTTTAGTGAGGC	61	Flot et al. (2011)
		ITSc1-3	CATTTGCGTTCAAAGATTCG	61	Flot et al. (2011)
ITS2 rDNA (nuclear)	*Stylophora*	ITSc2-5	AGCCAGCTGCGATAAGTAGTG	55	Flot and Tillier (2006)~~Flot and Tillier (2006)~~
		R28S1	GCTGCAATCCCAAACAACCC	55	Flot and Tillier (2006)
HSP70 (nuclear)	*Stylophora*	HSP70A-F	CCTGGTTCAATCCGACAGA	59	Schmidt-Roach et al. (2012)
		HSP70A-R	TGTTCAGCTGTTTTCCTTCG	59	Schmidt-Roach et al. (2012)
		HSP70B-F	CTATCCAGGCAGCGGTCTT	59	Schmidt-Roach et al. (2012)
		HSP70B-R	TGGTGAACACACTTGCTGTAG	59	Schmidt-Roach et al. (2012)
psbA (plastid)	*Symbiodinium*	psbAFor_1	GCAGCTCATGGTTATTTTGGTAGAC	64	LaJeunesse and Thornhill (2011)
		psbARev_1	AATTCCCATTCTCTACCCATCC	64	LaJeunesse and Thornhill (2011)

Figure S1. Mitochondrial haplonets of *Stylophora* morphospecies belonging to clade 4. (**A**) Control Region~~R~~, (**B**) Open Reading Frame~~RF~~. Each circle represents a haplotype and its size is proportional to its total frequency. Small grey circles represent missing haplotypes, small orange circles represent a single nucleotide change, and black cross-bars represent greater than one change (as indicated below them). Colours denote *Stylophora* species as indicated by the embedded key.

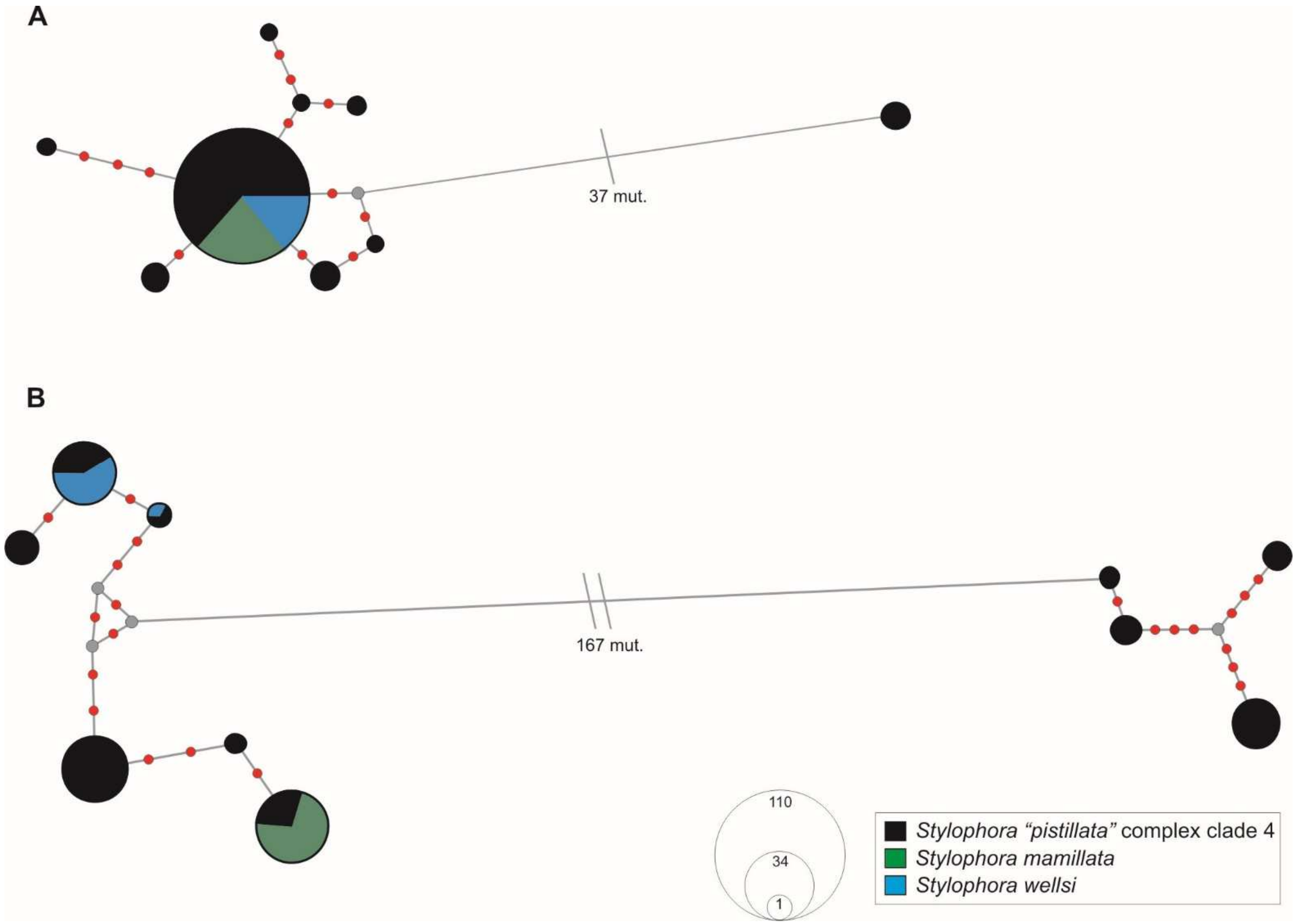

Figure S2. Bayesian phyogenetic reconstruction of the genus *Stylophora* based on the nuclear HSP70 gene. Values at branches represent posterior Bayesian probabilities (> 0.7), ML SH-like support (> 70%), and MP bootstrap values (> 70%), respectively. Dashes (-) indicate nodes that are statistically unsupported. Sequences obtained in this study are indicated in bold. Colours denote *Stylophora* species as indicated by the embedded key. Clade numbers refer to[20].

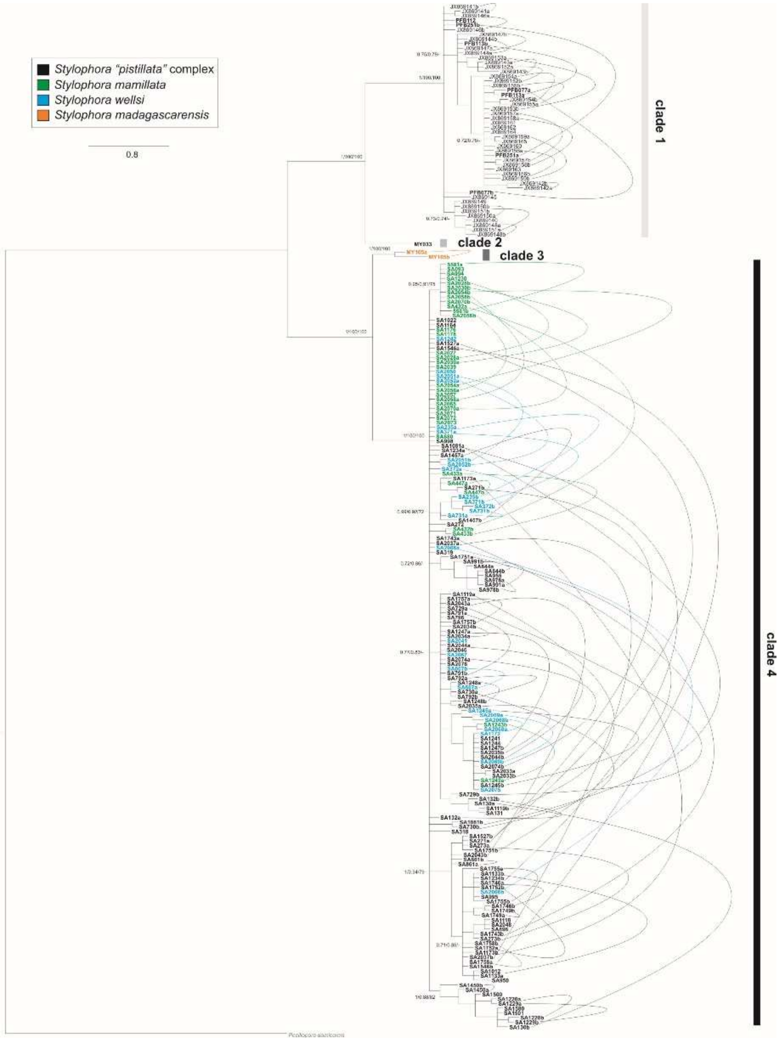

Supplement: Supplementary Information [file srep34612-s1.pdf]
